# Dapsone-induced agranulocytosis leading to perianal abscess and death: a case report

**DOI:** 10.1186/1752-1947-5-107

**Published:** 2011-03-16

**Authors:** Yoshiro Kobe, Daisuke Setoguchi, Nobuya Kitamura

**Affiliations:** 1 Department of Emergency and Critical Care Medicine, Kimitsu Chuo Hospital, Kisarazu, Chiba, 292-8535, Japan

## Abstract

**Introduction:**

Dapsone (diaminodiphenylsulfone) is used for the treatment of intractable skin diseases such as pemphigus and leprosy. The side effects of Dapsone are anemia, leukopenia, and liver dysfunction. Here, we present a case of agranulocytosis-induced septic shock, which was a side effect of Dapsone.

**Case presentation:**

An 82-year-old Japanese woman was transferred to our hospital with fever, leucopenia, and respiratory arrest. At the previous hospital, she had been administered Dapsone for linear IgA bullous dermatosis. At the time of admission, she presented with methemoglobinemia and septic shock, which was due to immunosuppression caused by the normal dose of Dapsone. Although her overall health initially improved, her condition deteriorated because of septic shock caused by an anal fistula. She died of sepsis on hospital day 80.

**Conclusion:**

One of the side effects of Dapsone is agranulocytosis. Patients with agranulocytosis may be in danger of developing anal fistula. Therefore, care must be taken if a patient with agranulocytosis develops a decubitus ulcer in the sacral region, since it could develop into a fistula-in-ano.

## Introduction

Dapsone (diaminodiphenylsulfone) has been used for treating intractable skin diseases such as leprosy and dermatitis herpetiformis. The side effects associated with the use of Dapsone include hemolytic anemia, methemoglobinemia, and agranulocytosis [1]. Agranulocytosis is a rare condition; however, it can become a life-threatening illness if sepsis develops.

We report a case of agranulocytosis as a side effect of Dapsone, which was administered to a patient for treating linear IgA bullous dermatosis (LABD). Agranulocytosis-induced septic shock and perianal abscess occurred, and the patient died from sepsis and multiple organ failure.

## Case presentation

An 82-year-old Japanese woman was transferred to our emergency room with respiratory arrest and leukocytopenia. She had previously been admitted to a hospital with high fever (38 to 40°C) and was treated with antibiotics for six days before being admitted to our hospital. She had diabetes mellitus, hyperlipidemia, and hypertension, and she took prednisolone (15 mg/day) and Dapsone (75 mg/day for seven days and 100 mg/day for about six weeks) for LABD. Her physical examination, which was conducted at our hospital, revealed the following: cold clammy skin; no jugular venous distention; no edema; no apparent skin lesions, which suggested good response for LABD with Dapsone; nonresponsiveness despite her eyes being open (Glasgow Coma Scale score of 10, E4 V1 M5). Her blood pressure was 100/54 mm Hg; pulse rate, irregular and tachycardia (150 beats/minute); respiratory rate, 14 beats/minute; SpO_2_, 89% (pulse oximetry, 6 L/minute O_2 _under intubation); and body temperature, 38.3°C. No rales were heard on auscultation.

The initial laboratory tests (Table 1) revealed a white blood cell count of 400/μL (reference range: 4000 to 10,000 cells/μL); neutrophil count, 8 cells/μL (reference range: 2000 to 7000 cells/μL); hemoglobin, 7.6 g/dL (reference range: 12.0 to 16.0 g/dL) and platelet count, 183 × 10^3^/μL (reference range: 140 to 450 × 10^3^/μL). The results of coagulation studies were normal. Serum chemistry showed elevated total bilirubin level, 3.0 mg/dL (reference range: 0.2 to 1.2 mg/dL); glucose, 306 mg/dL (reference range: 70 to 110 mg/dL) and C-reactive protein (CRP), 21.6 mg/dL (reference range: below 0.3 mg/dL). The low level of hemoglobin and high level of bilirubin were indicative of hemolytic anemia, whereas no hemolysis was shown in peripheral smear. Further, the results of arterial blood gas (ABG) analysis, under supplementation of 100% O_2_, revealed the following: pH, 7.51; pCO_2_, 30 mm Hg; pO_2_, 415 mm Hg; base excess, 1.0 mmol/L; lactate, 2.2 mmol/L (reference range: below 1.3 mmol/L) and Methemoglobin, 9.0% (reference range: below 3.0%).

**Table 1:** Laboratory investigations on admission to Kimitsu Chuo Hospital Intensive Care Unit

Complete blood count		Arterial blood gas (F_I_O_2 _1.0)	
WBC	0.4 × 10^3^/μL	pH	7.51
RBC	2.25 × 10^6^/μL	pCO_2_	30 mmHg
Hgb	7.6 g/dL	pO_2_	415 mmHg
Hct	22.8%	HCO_3_	25.8 mmol/L
Plt	183 × 10^3^/μL	BE	1.0 mmol/L
Reticulo	27.8%	Lactate	2.2 mmol/L
		Met Hgb	9.0%

**Coagulation studies**		**Rapid Urinary Antigen Detection Kit**	

PT INR	1.19	*Streptococcus pneumoniae*	negative
aPTT	26.9 s	*Legionella*	negative

**Serum Chemistry**			

Alb	2.1 g/dL	Na	129 mEq/L
AST	17 IU/L	K	4.4 mEq/L
ALT	22 IU/L	Cl	96 mEq/L
LDH	247 IU/L	BUN	18.7 mg/dL
T-bil	3.0 mg/dL	Cre	0.55 mg/dL

WBC: White blood cell; RBC: Red blood cell; Hgb: Hemoglobin; Hct: Hematocrit; Plt: Platelet; Reticulo: Reticulocyte; PT INR: Prothrombin time international normalized ratio; aPTT: Activated partial thromboplastin time; Alb: Albumin; AST: Asparatate transaminase; ALT: Alanine transaminase; LDH: Lactate dehydrogenase; T-bil: Total bilirubin; BUN: Blood urea nitrogen; Cre: Creatinine; HCO_3_: Bicarbonate; BE: base excess; Met Hgb: Methemoglobin.

Computed tomography (CT) scans of the head revealed no intracranial abnormalities. Chest X-ray images revealed no infiltration. An electrocardiography (ECG) scan revealed tachycardia (152/minute) with atrial fibrillation. Cultures of blood, sputum, and urine samples collected at the time of admission were negative for fungal or bacterial growth. *Streptococcus pneumoniae *and *Legionella *were found to be absent in the urine samples with the rapid urinary antigen detection kit. Cerebrospinal fluid from a lumbar puncture was negative for bacteria and fungi.

**Figure 1:**
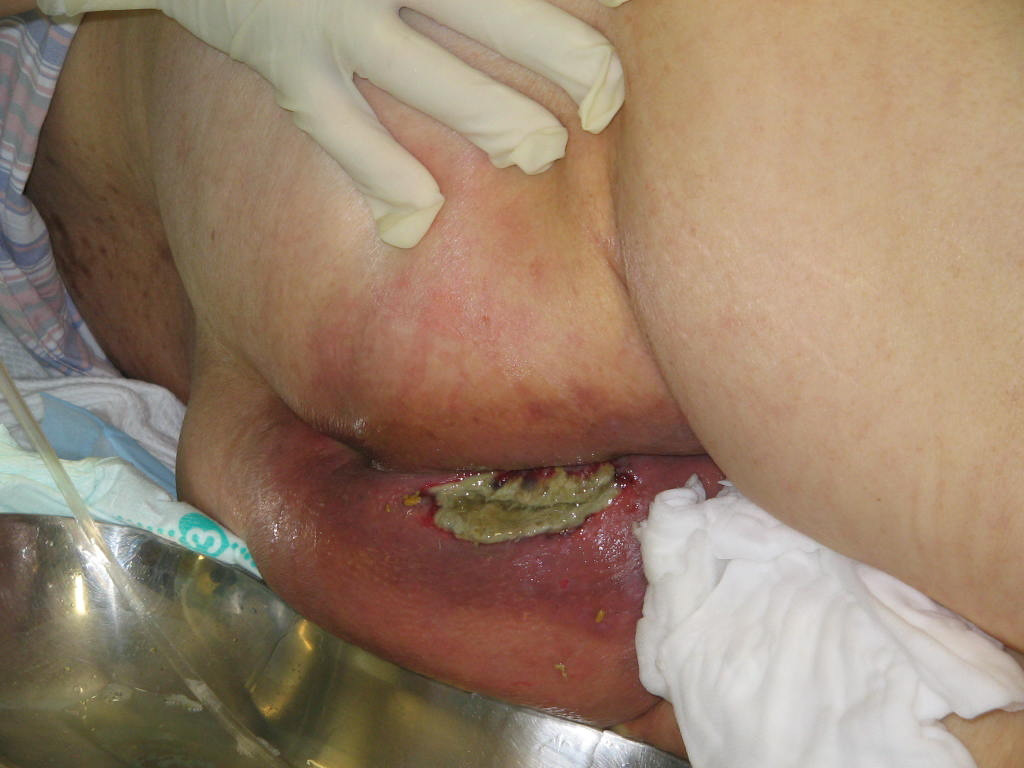
**The appearance of fistula-in-ano at the early stage**.

**Figure 2:**
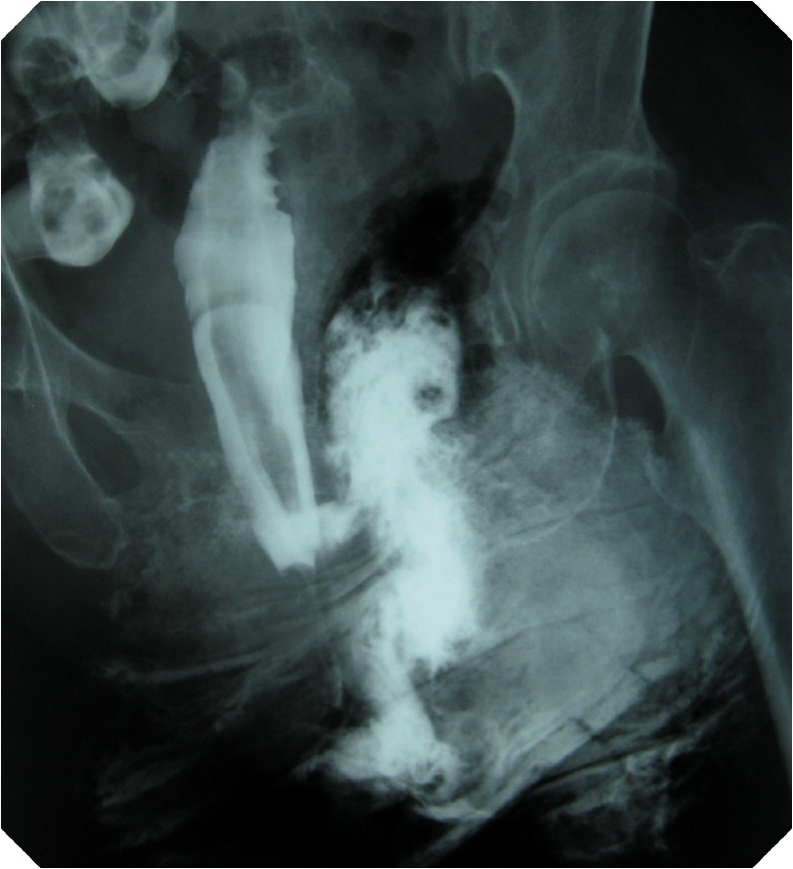
**Fistulogram showing a fistula communicating between the rectum and the postsacral region**.

**Figure 3:**
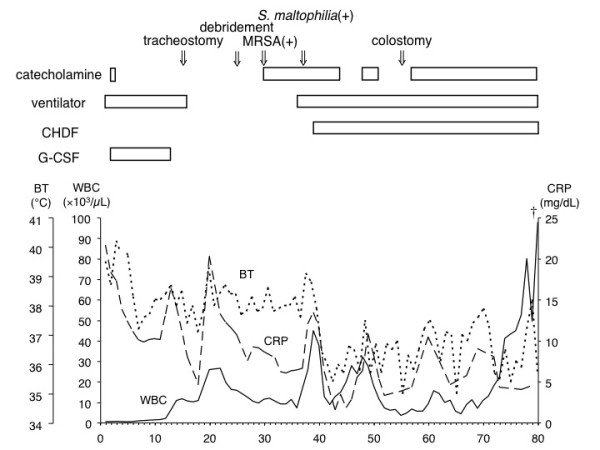
**Clinical course of a patient with Dapsone-induced agranulocytosis**. BT: Body temperature; CHDF: Continuous hemodiafiltration; CRP: C-reactive protein; G-CSF: Granulocyte colony-stimulating factor; MRSA: Methicillin-resistant *Staphylococcus aureus*; WBC: White blood cell

Although the cause of agranulocytosis was initially unknown, we later found that the patient was taking Dapsone for LABD. On the basis of this finding, we deduced that the agranulocytosis was induced by Dapsone, which also induced hemolytic anemia and methemoglobinemia, and that agranulocytosis was responsible for her septic shock. She was subsequently admitted to the intensive care unit (ICU) and received mechanical ventilation. Meropenem and fosfluconazole were administered intravenously. The levels of methemoglobinemia decreased to 3% because Dapsone was discontinued; therefore, no treatment for methemoglobinemia was required. On Day 1 after being admitted to our hospital, her leukocyte count was lower than 400/μL; however, after granulocyte-colony stimulating factor (G-CSF) treatment was initiated, the leukocyte count increased to 6100/μL on Day 13. Mechanical ventilation was discontinued on Day 10 because of stabilization of her circulatory and respiratory status. However, on Day 13 gradual exacerbation of pneumonia caused her reintubation and initiation of mechanical ventilation and tracheostomy was performed on Day 15. Postsacral erosion and induration appeared on Day 18. On the same day, her body temperature was >39°C and atrial fibrillation occurred (heart rate, >150/minute). Laboratory tests revealed a white blood cell count of 26,000/μL and CRP level of 13 to 16 mg/dL. This was indicative of persistent inflammation. The postsacral region was incised, and her decubitus ulcers were drained because exudates with the smell of feces were discharged from the postsacral region. The region was necrotized to a depth of 5 cm, and the drainage materials were found to be feces (Figure 1). A contrast fistulogram revealed the presence of a fistula joining the rectum to the postsacral region (Figure 2). Inflammation was persistent, and her body temperature increased to 39°C on Day 30 despite repeated, almost daily, lavage, debridement, and administration of sulfadiazine silver for the fistula. Linezolid was administered intravenously for suspected sepsis caused by methicillin-resistant *Staphylococcus aureus *(MRSA). MRSA was later identified in blood and central venous catheter tip cultures. The blood culture was persistently positive for *Stenotrophomonas maltophilia *after Day 37, and she developed septic shock once again along with renal failure. Her general status temporarily improved with continuous hemodiafiltration (CHDF) and administration of catecholamines. Although colostomy was performed on Day 55, she died on Day 80 because of persistent shock and gastrointestinal hemorrhage (Figure 3).

## Discussion

Dapsone has been used for treating leprosy since the 1940s, and a few dermatological disorders of autoimmune origin since the 1950s [1,2]. It is also effective for dermatological conditions such as dermatitis herpetiformis, LABD, bullous pemphigoid, pemphigus, and erythema elevatum diutinum. Dapsone also has antibacterial and anti-inflammatory effects. The mechanism underlying the former is the inhibition of bacterial synthesis of folate; however, the mechanism underlying the anti-inflammatory effect is unknown [2]. The recommended dose of Dapsone is 50 to 100 mg. Usually, serious side effects are not observed when the dose is <100 mg/day; however, they do occur when the dose is >200 mg/day. Moreover, it is recommended that the dose should not exceed 300 mg/day [2].

The most common side effects of Dapsone are hemolytic anema, methemoglobinemia, and agranulocytosis [1]. Hemolytic anemia is a dose-dependent side effect that usually occurs three to four weeks after Dapsone therapy is initiated if the dose is >300 mg/day [2]. Although the mechanism underlying the side effects is unknown, it has been reported that Dapsone reduces the lifespan of red blood cells [2]. The level of methemoglobinemia increases in a dose-dependent manner, especially in infants and elderly patients. The common symptoms of methemoglobinemia are headache, dyspnea, and tachycardia. Careful observation is required when the percentage of methemoglobin is approximately 10 to 20% and if a patient does not exhibit the above mentioned symptoms. Dapsone therapy should be discontinued for patients with abnormal cardiopulmonary function when the percentage of methemogloblin is 5%. The fundamental treatment for methemogloblinemia is careful observation. Severe cases of methemoglobinemia, however, can be treated with intravenous methylene blue (1 to 2 mg/kg). In a well-known study, it was reported that agranulocytosis developed in 16 US soldiers in Vietnam who were receiving Dapsone prophylaxis for falciparum malaria [3]. Agranulocytosis develops 4 to 12 weeks after Dapsone therapy is initiated, and it gradually progresses. The initial symptoms are fever, swelling of the lymph nodes, and inflammation and ulcers of the oral cavity, pharynx, and esophagus. Once agranulocytosis develops, a patient's increased susceptibility to sepsis and death may occur. However, unlike methemoglobinemia, agranulocytosis is not a dose-dependent side effect of Dapsone, and the mechanism of agranulocytosis due to Dapsone remains unknown.

In our case, the cause of shock along with agranulocytosis was initially unknown. Methemoglobinemia was recognized because the patient's PaO_2 _level was high despite the low SpO_2 _level. We found that she had taken Dapsone, a side effect of which was methemoglobinemia, so we diagnosed her with septic shock due to Dapsone-induced agranulocytosis [4]. Although the patient took only 100 mg per day for 45 days, she developed severe agranulocytosis as a side effect. In the 1970s and 1980s, there were many studies that reported the side effects of Dapsone, and almost all these studies reported that Dapsone-induced side effects occurred when a few hundred milligrams to a few grams of Dapsone were administered to patients. However, it was also reported that 50 to 125 mg of Dapsone induced severe agranulocytosis. Patients taking Dapsone for dermatitis herpetiformis are at a 25- to 33-fold greater risk of agranulocytosis than normal [1]. Careful observation is required when treating with Dapsone for an inflammatory disease such as LABD. Her hemoglobin prior to initiation of Dapsone therapy was 13.7 g/dL. No blood tests were performed during Dapsone therapy because of the normal hemoglobin level. In order to monitor anemia, leukocytopenia, and methemoglobinemia, blood tests are required every week for the first month, and only every two weeks to every month thereafter, even if patients are administered 100 mg/day of Dapsone.

The patient recovered once from septic shock, but she eventually died of multiple organ failure due to recurrent sepsis from an anal fistula that developed from a decubitus ulcer of the sacral region. In most cases, the causes of fistula-in-ano are radiation therapy and surgical complication. Anal fistula is a frequent complication of leukemia and agranulocytosis. It has been reported that 8 to 60% of leukemia patients develop perianal and perirectal infection; however, the exact figure is unknown [5,6]. To our knowledge, Dapsone-induced agranulocytosis that develops to fistula-in-ano has not been previously reported. Although the reason patients with leukemia and agranulocytosis tend to develop fistula-in-ano is unknown, it is speculated that decubitus, which is caused by prolonged immobility, develops into an abscess because of dysfunction; when this is accompanied by a decrease in the number of granulocytes, which are responsible for immune response, the decubitus develops into an anal fistula [7]. The treatment approach for fistula-in-ano is appropriate antibiotic administration and incisional drainage, provided that background diseases such as agranulocytosis and leukemia can be controlled and the patient's overall condition permits it [8,9]. Sepsis-associated fistula-in-ano increases the mortality rate even if incisional drainage is properly performed. In our case, we performed incisional drainage soon after the diagnosis of fistula-in-ano was made; it was followed by antibiotic administration and several debridements. The patient died of sepsis without resolution of the anal fistula; however, we could have performed the incisional drainage earlier if we could have predicted the development of the decubitus ulcer in the sacral region into the anal fistula and if we had conducted a rectal examination. The development of fistula-in-ano should always be considered for patients with Dapsone-induced agranulocytosis, and it is necessary to examine for infection around the rectum when a decubitus ulcer is formed in the sacral region.

## Conclusion

We report the case of a patient with Dapsone-induced agranulocytosis that progressed to septic shock. She temporarily recovered from septic shock but eventually died when the infection could not be controlled. Incisional drainage was performed for anal fistula, which developed from a decubitus ulcer in the sacral region. Agranulocytosis is one of the side effects of Dapsone; therefore, it should be administered with care. Further, for patients with agranulocytosis, if a decubitus ulcer is found in the sacral region, it should be monitored carefully because it may develop into fistula-in-ano.

## Consent

Written informed consent was obtained from the patient's daughter for publication of this case report and accompanying images. A copy of the written consent is available for review by the Editor-in-Chief of this journal.

## Competing interests

The authors declare that they have no competing interests.

## Authors' contributions

YK wrote the case report, conducted the literature search and obtained the consent. DS contributed to the discussion. NK supervised and edited the case report. All authors were involved with treatment of this patient and all read and approved the final manuscript.
